# Pruning deficits of the developing *Drosophila* mushroom body result in mild impairment in associative odour learning and cause hyperactivity

**DOI:** 10.1098/rsob.220096

**Published:** 2022-09-21

**Authors:** Haiko Poppinga, Büşra Çoban, Hagar Meltzer, Oded Mayseless, Annekathrin Widmann, Oren Schuldiner, André Fiala

**Affiliations:** ^1^ Department of Molecular Neurobiology of Behaviour, University of Göttingen, Julia-Lermontowa-Weg 3, 37077 Göttingen, Germany; ^2^ Departments for Molecular Cell Biology and Molecular Neuroscience, Weizmann Institute of Science, Ullmann Building of Life Sciences, Rehovot 7610001, Israel

**Keywords:** *Drosophila melanogaster*, mushroom body, Kenyon cells, neuronal remodelling, associative learning, circadian rhythm

## Abstract

The principles of how brain circuits establish themselves during development are largely conserved across animal species. Connections made during embryonic development that are appropriate for an early life stage are frequently remodelled later in ontogeny via pruning and subsequent regrowth to generate adult-specific connectivity. The mushroom body of the fruit fly *Drosophila melanogaster* is a well-established model circuit for examining the cellular mechanisms underlying neurite remodelling. This central brain circuit integrates sensory information with learned and innate valences to adaptively instruct behavioural decisions. Thereby, the mushroom body organizes adaptive behaviour, such as associative learning. However, little is known about the specific aspects of behaviour that require mushroom body remodelling. Here, we used genetic interventions to prevent the intrinsic neurons of the larval mushroom body (γ-type Kenyon cells) from remodelling. We asked to what degree remodelling deficits resulted in impaired behaviour. We found that deficits caused hyperactivity and mild impairment in differential aversive olfactory learning, but not appetitive learning. Maintenance of circadian rhythm and sleep were not affected. We conclude that neurite pruning and regrowth of γ-type Kenyon cells is not required for the establishment of circuits that mediate associative odour learning *per se*, but it does improve distinct learning tasks.

## Graphical abstract

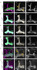

## 1. Background

Neuronal circuits establish functional connections during development in animal brains. Initially, synaptic connections form in excess. Subsequent regressive pruning of dendrites and axons eliminates many of these connections, and neurite regrowth establishes new, adult-specific connections [1]. The cellular and circuit mechanisms underlying localized degeneration and regeneration of axons and dendrites are the subject of intense research. In this context, the mushroom body of the *Drosophila* brain is a favourable and widely studied neuronal circuit [2–5]. Sophisticated genetic tools available for this model organism permit the manipulation and visualization of distinct neurons with high precision [6]. As a further advantage, the process of mushroom body remodelling during metamorphosis is genetically determined and stereotypic across individuals. Moreover, the exact synaptic connectivities of the neurons that constitute larval and adult mushroom bodies have been characterized in detail [7–10]. The behavioural functions of the *Drosophila* mushroom body are also well studied [11,12]. A diverse repertoire of behaviours are under mushroom body control, including associative olfactory [12] and visual learning [13], courtship conditioning [14], locomotor control [15], sleep [16] and food uptake [17,18].

The mushroom body consists of intrinsic neurons called Kenyon cells (KC) that receive sensory input at the main dendritic compartment, the calyx. Parallel axons collectively form the lobes of the mushroom body. In terms of cell number, the mushroom body of the larval brain is simpler than that of the adult fly. During development, a larval mushroom body is generated, consisting of approximately 650 bifurcated KCs at the third larval stage, giving rise to a medial and a vertical lobe. However, the mushroom body circuitry is already complex at the larval stages. Extrinsic mushroom body neurons synapsing onto KCs include sensory input neurons, dopaminergic neurons that transmit valence-signalling information (also called mushroom body input neurons, MBINs), mushroom body output neurons (MBONs) that guide appropriate locomotor behaviour, one broadly ramifying GABAergic inhibitory neuron, and neurons that release neuromodulators, e.g. octopamine and serotonin [19]. During metamorphosis the larval KCs (γ-KCs) survive, but their dendrites and axons undergo massive remodelling (i.e. pruning and regrowth). These embryonic-born γ-KCs regrow their axons with only one axonal branch, forming the medially projecting, adult γ-lobe [3–5]. During the late larval and pupal stages, additional KCs are born with bifurcating axons that form the medial *β* and *β′* lobes, and the vertical *α* and *α′* prime lobes [20], resulting in approximately 2000 KCs per brain hemisphere. How the remodelling of γ-KCs contribute to the function of the adult mushroom body is not known.

In summary, the connectivity of an already-complex larval mushroom body is replaced by a numerically more complex adult mushroom body that has, in addition to embryonic-born γ-KCs, larval-born *α′*/*β′*-KCs and pupal-born *α*/*β*-KCs. However, little is known about how mushroom body remodelling influences behavioural control, nor why γ-KCs undergo drastic remodelling rather than persist. In this study, we investigated behavioural deficits in animals after the prevention of neurite pruning and regrowth of γ-KCs. It has already been shown that altered remodelling of KCs results in short-term memory impairments in courtship conditioning, an adult-specific learning regime in which male flies learn to avoid unsuccessful courtship attempts; Long-term memory remains unaffected [21]. Here, we first investigated whether circadian locomotor activity over several days depends on proper mushroom body development. Second, we tested the animals in a widely used associative olfactory learning paradigm [22,23]. We were particularly interested in this type of learning because both larvae and adult flies can learn to associate odours with appetitive or aversive cues [12,24]. It is also well known that the initial associative learning process and short-term memory recall depend on γ-KCs in adults [25,26], and on embryonic γ-KCs in larvae [24]. However, it is unknown whether remodelling of γ-KCs is required for adults to establish a functional circuit to learn properly, or whether persistent embryonic γ-KCs and a preserved larval circuit architecture can mediate associative learning in adults. Therefore, we asked whether embryonic-born and remodelled adult γ-KCs are functionally equivalent.

## 2. Material and methods

### 2.1. Fly strains

Flies were raised on regular cornmeal medium at 25°C, under 65% relative humidity and a 12 h/12 h light-dark cycle, unless indicated otherwise. The following fly strains were used: GMR71G10-Gal4 [27] which drives Gal4 predominantly in γ-KCs, UAS:EcR-B1^W650A^ [28] expressing a dominant-negative form of the ecdysone receptor, also known as UAS:EcR^DN^, and UAS:Tai^DN^ [29], which expresses a dominant-negative variant of the ecdysone receptor coactivator Taiman fused with GFP. The w^1118^ strain used was obtained from BestGene Inc (Chino Hills, CA, USA).

### 2.2. Analysis of locomotion, sleep and circadian rhythm

Individual, 3-day-old male flies were transferred to glass tubes (5-mm diameter, 6.5-cm length). One end of each tube was filled with fly food and sealed with Parafilm. The opposite end was closed with an air-permeable plug. The flies' locomotion was monitored using the Drosophila Activity Monitor (DAM) system from Trikinetics [30]. The DAM recorder was kept in a humidity and temperature-controlled incubator under a 12 h/12 h light-dark cycle for 3 days, and for additional 5 days under constant darkness. Data were analysed using the ‘Sleep and Circadian Analysis MATLAB Program 2019_v2’ from Christopher G. Vecsey (Skidmore College, USA).

### 2.3. Associative learning

Groups of approximately 100 flies (3–5 days old) were trained as described in [23], with some modifications; i.e. four experiments were performed simultaneously in a modified learning apparatus [31,32]. Briefly, flies were conditioned to associate odours with an aversive or appetitive stimulus and then tested for odour preference in a T-maze assay. Each training tube had a constant airflow of approximately 167 ml min^−1^, assuring a constant odour flow inside. Before the onset of each experiment, flies were transferred to empty vials and kept for 10 min at the respective temperature. The odours 4-methylcyclohexanol and 3-octanol (Sigma-Aldrich) were diluted 1 : 100 in.mineral oil. Training started 1 min after transferring the flies into training tubes that were covered on the inside with an electrifiable grid or with Whatman paper. Each odour was presented for 1 min, with a 1 min break between the two odour applications. In the case of aversive conditioning, one odour (conditioned stimulus +, CS+) was temporally paired with 12 electric shocks of 90 V DC (1.25 s shock duration and 3.75 s inter-pulse interval). The second odour (conditioned stimulus, CS−) was presented without shocks. In the case of appetitive conditioning, flies were starved prior to training for 24 h by placing them in empty food vials with tissue paper soaked in 10 ml tap water at the bottom, and the CS+ odour was temporally paired with the presentation of sugar. This was achieved by using a tube designed such that a piece of Whatman paper soaked with a 2 M sucrose solution could be shifted by rotation to the inner site of the tube. The second odour (CS−) was presented without the sugar reward. After another minute, the flies were transferred to the T-maze part of the apparatus with both odours presented from each side, and flies were tested for their odour preference for 2 min. The flies were then counted, and a preference index was calculated by subtracting the number of flies on the side associated with the CS− from the number of flies on the side with the CS+, divided by the total number of flies. A learning index was calculated by averaging preference indices from two reciprocal experiments.

### 2.4. Odour preference

Three- to five-day-old flies were starved by placing them in empty food vials with tissue paper soaked in 10 ml tap water at the bottom. After 24 h, flies were transferred to the olfactory training apparatus, but not subjected to associative training. After 3 min of rest, the animals were moved to the T-maze choice point between two test tubes. The flies were allowed to distribute in either tube for 2 min. One tube was perfused with odour; the opposite tube was perfused with the solvent mineral oil. The number of flies in either tube was counted. An odour preference index was calculated as the number of flies in the tube with the odour minus that in the opposite tube, divided by the total number of flies.

### 2.5. Electric shock avoidance

3- to 5-day-old flies were transferred into tubes covered with electric grids and positioned in the T-maze part of the training apparatus. After 3 min of rest, the animals were allowed to distribute for 2 min between two tubes, one of which was electrified every 5 s for 1.25 s, resulting in 24 electric shocks. A preference index was calculated as the number of flies in the electrified tube minus the number in the opposite tube, divided by the total number of flies.

### 2.6. Immunohistochemistry

*Drosophila* brains were dissected in cold ringer solution and fixed using 4% paraformaldehyde for 20 min at room temperature (25°C) on a nutator. Subsequently, the brains were washed in phosphate buffer with 0.3% Triton-X (PBT; 3 × immediate washes followed by 3 × 20 min washes), blocked using 5% heat-inactivated goat serum in PBT, and incubated overnight at 4°C with primary antibodies (chicken anti-GFP, AVES: GFP-1020, 1:500; mouse monoclonal anti-FasII, Developmental Studies Hybridoma Bank (DSHB): 1D4, 1:25; mouse monoclonal anti-Trio, DSHB: 9.4A, 1:50). After three immediate and three 20 min washing steps with PBT, the brains were incubated with secondary antibodies for 2 h at room temperature (FITC-coupled goat anti-chicken, Invitrogen: A-16055, 1:300 and Alexa fluor-647-coupled goat anti-mouse, Invitrogen: A-32728, 1:300). Secondary antibodies were washed off using PBT prior to mounting (3 × immediate and 3 × 20-min washes). Brains were mounted on Slowfade (S-36 936; Invitrogen) and imaged using a Zeiss LSM 800 confocal microscope with a 40 × 1.3 NA oil immersion lens. Images were processed with ImageJ.

### 2.7. Statistical analysis

Statistical tests were performed using OriginPro 8.50 software (OriginLab). The Kolmogorov–Smirnov test was used to determine whether the data were normally distributed. Significant differences between groups were tested using one-way ANOVA and Bonferroni *post-hoc* tests.

## 3. Results

**Figure 1.**
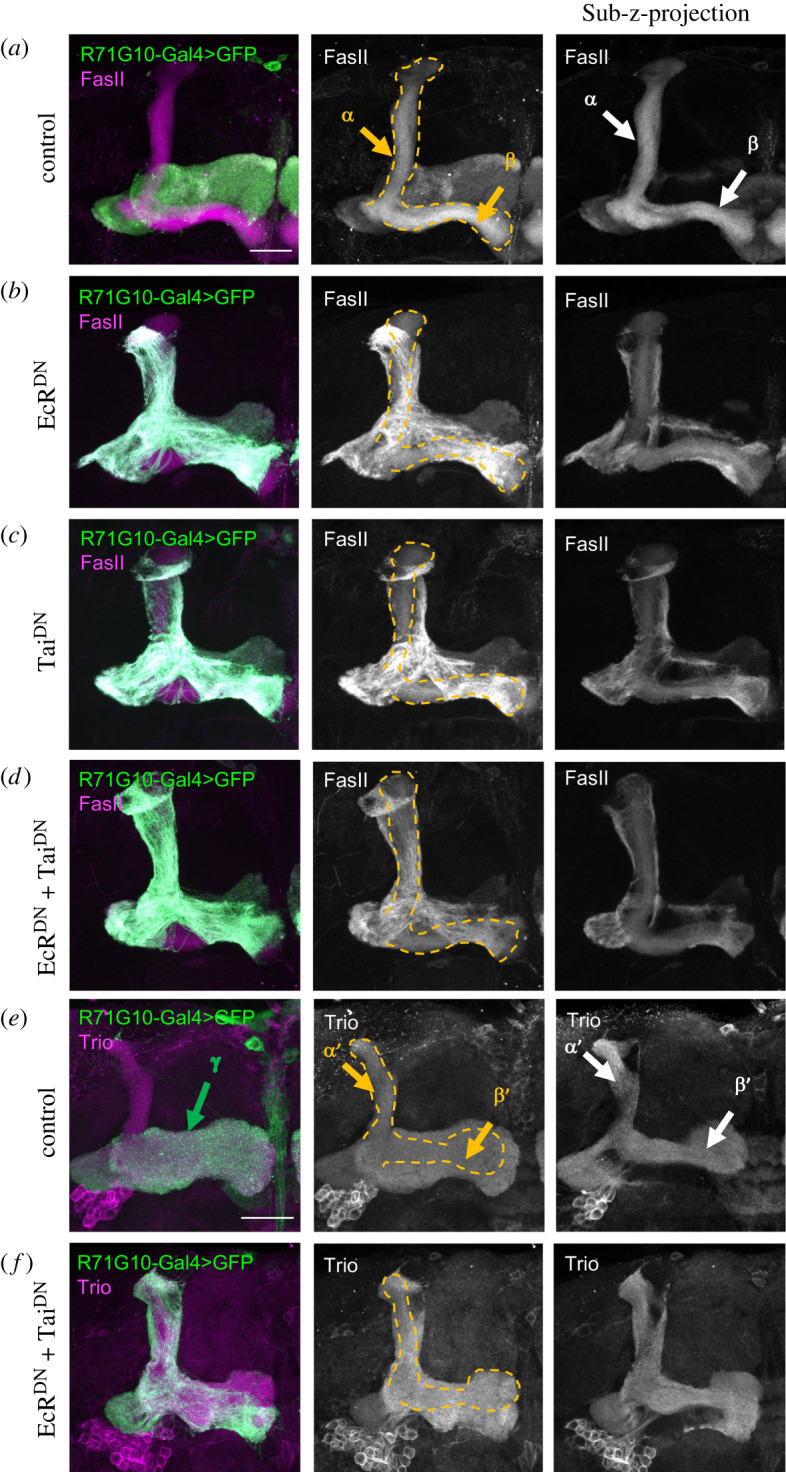
Co-expressing dominant negative forms of EcR and Tai in *γ* neurons using R71G10-Gal4 blocks pruning of mushroom body (MB) *γ* neurons, but does not affect the development of the *α/β* or *α′/β′* KCs. Confocal Z-projections of the axonal MB region in brains of animals with different genotypes. The left rows show in green membrane-bound GFP driven by 71G10-Gal4, and in magenta antibody staining against either FasII (which strongly labels *α*/*β* neurons, weakly labels *γ* neurons and does not stain *α*′/*β*′ neurons (*a*–*d*)) or Trio (which strongly labels *α*′/*β*′ neurons, as well as *γ* neurons, but does not stain *α*/*β* neurons (*e*,*f*)). The middle row shows the antibody staining only. The right row shows the antibody staining in a sub-projection of fewer confocal planes focused on the *α*/β-lobes (*a*–*d*) or *α*′/*β*′-lobes (*e*,*f*). (*a*) Wildtype (control) with anti-FasII staining. MB *γ* axons collectively form the unbranched medial *γ* lobe (*γ*). Upon 71G10-Gal4 driven expression of a dominant negative ecdysone receptor (EcR^DN^) (*b*) or dominant negative Tai (Tai^DN^) (*c*) branched, larval *γ* neurons persist due to failed pruning. A combination of both transgenes (*d*) even slightly enhances the pruning deficit, which is visualized as the near complete lack of adult-specific projections. Note that the shape of the *α/β*-lobes (*α* and *β*), outlined as yellow dashed lines in the middle row, remains intact in all genotypes. (*e*) Wildtype (control) with anti-Trio staining. MB *γ* axons collectively form the unbranched medial *γ* lobe (*γ*). (*f*) Upon 71G10-Gal4 driven expression of a dominant negative ecdysone receptor (EcR^DN^) and dominant negative Tai (Tai^DN^) branched, embryonic *γ* neurons persist due to failed pruning. Note that also the shape of the *α*′/*β*′-lobes (*α*′ and *β*′), outlined as yellow dashed lines in the middle row, remains intact in all genotypes. Scale bar: 30 µm.

Axonal and dendritic pruning of γ-KCs was prevented by expressing a dominant-negative variant of the ecdysone receptor (EcR^DN^) [28] together with a dominant-negative variant of the ecdysone receptor coactivator Taiman (Tai^DN^) [29] under control of the driver line GMR71G10-Gal4 [27]. This Gal4-line drives predominantly adult γ-KCs as well as along development (figure 1). Leaky expression in sparsely distributed *α*/β-KCs in adults has also been observed and cannot be excluded. The gross morphology of the *α*/β- (figure 1*a–d*) and *α*′/*β*′-lobes (figure 1*e,f*) appeared normal. By contrast, the γ-lobes were largely unpruned as evident by vertical axons running in parallel to the α- and *α*′-lobes, as in larvae, and the horizontally projecting axons of γ-KCs that maintained their larval location. Both the ectopic expression of EcR^DN^ and Tai^DN^ alone caused strong pruning defects, as demonstrated previously [33] (figure 1*b,c*), but the penetrance of the pruning-inhibiting effect was even enhanced by co-expressing both (figure 1*d,f*). Thus, combined expression of EcR^DN^ and Tai^DN^ results in the most robust and severe pruning defect that we have analysed to date, with a seemingly complete lack of the stereotypically located adult medial γ-lobe.

**Figure 2.**
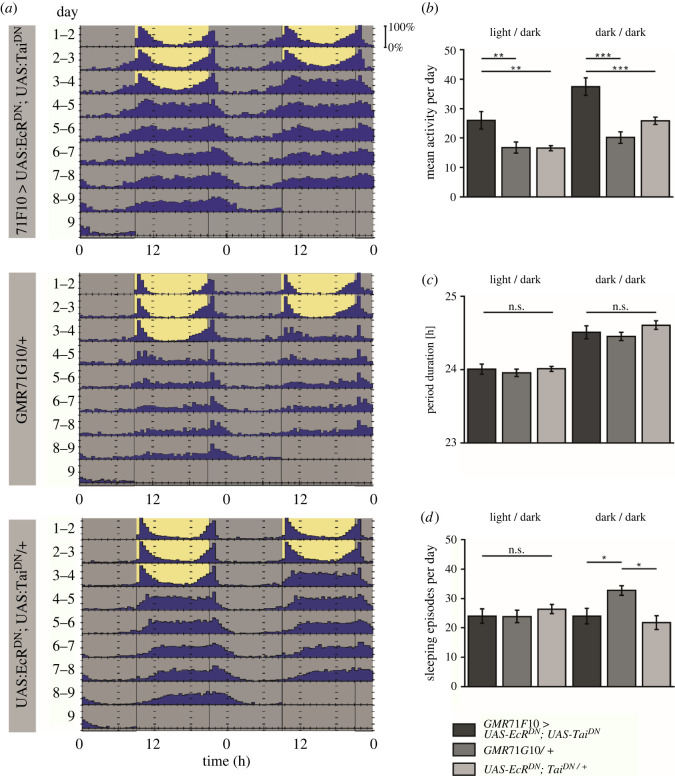
Pruning deficits of γ-KCs cause hyperactivity. (*a*) The first 3 days the flies were kept under a 12 h/12 h light-dark cycle (indicated by the yellow/grey background), followed by complete darkness (indicated by grey background) for 5 days. Each black bar shows activity counts per hour, reflecting the mean of activity counts of all flies within each group (*n* = 17, 20, 20). The x-axis indicates the real time in hours. The light/dark cycle matched the light/dark cycle under which the flies were raised. (*b*) The experimental group (GMR71G10-Gal4 > UAS-EcR^DN^; UAS-Tai^DN^) showed significantly stronger locomotor activity compared with the heterozygous parental strains (GMR71G10 > UAS-EcR^DN^; UAS-Tai^DN^: 26.02 ± 2.92, GMR71G10/+: 16.78 ± 1.85, UAS-EcRDN; TaiDN/+: 16.61 ± 0.86); especially in the 24 h dark cycle where the average activity was almost doubled (GMR71G10 > UAS-EcR^DN^; UAS-Tai^DN^: 37.53 ± 2.99, GMR71G10/+: 20.17 ± 1.95, UAS-EcR^DN^; UAS-Tai^DN^/+: 25.90 ± 1.21). (*c*) The period of the circadian rhythm was measured during the 12 h/12 h light/dark and 12 h/12 h dark/dark cycle. The period for all groups was close to 24 h (24.01 ± 0.07 h; 23.96 ± 0.05 h; 24.01 ± 0.03 h), showing that the flies adapted to the light/dark cycle. During the dark/dark cycle the period duration increased slightly (24.50 ± 0.09 h, 24.45 ± 0.06 h, 24.61 ± 0.06 h) but was not significantly different between the groups. (*d*) The number of sleeping episodes during the light/dark cycle was 24 ± 2.44 for the experimental group and was not significantly different from that of the parental control groups (23.87 ± 2.15; 26.42 ± 1.56). In the dark/dark cycle the GAL-4 line had a significantly increased number of sleeping episodes (32.73 ± 1.62) compared to the experimental (23.99 ± 2.61) and the other parental control group (21.81 ± 2.37). Bars indicate means ± SEM. *p < 0.05; **p < 0.01; ***p < 0.001.

Male flies of this genotype were subjected to a test of walking activity over 8 consecutive days using a locomotor assay [30]. Briefly, we counted the number of times that flies kept in small glass tubes crossed an infrared beam over time. We found that impairing remodelling of γ-KCs resulted in increased locomotion during a 12 h light/dark regime compared with heterozygous parental control strains that carried either the UAS constructs or the Gal4 construct only (figure 2*a,b*). This hyperactivity was even more pronounced during a subsequent 24 h dark cycle (figure 2*a,b*). However, the overall circadian rhythm was not affected, as evidenced by the finding that the period of rhythmic locomotor activity was similar between the three genotypes at around 24 h under light-entrained conditions, and slightly higher in complete darkness (figure 2*c*). Sleep, commonly defined in *Drosophila* as periods of inactivity lasting at least 5 min [34], was also not affected (figure 2*d*). The parental Gal4 strain showed slightly but statistically significant more sleep episodes, but not the flies with induced pruning deficits or the UAS strain (figure 2*d*).

**Figure 3.**
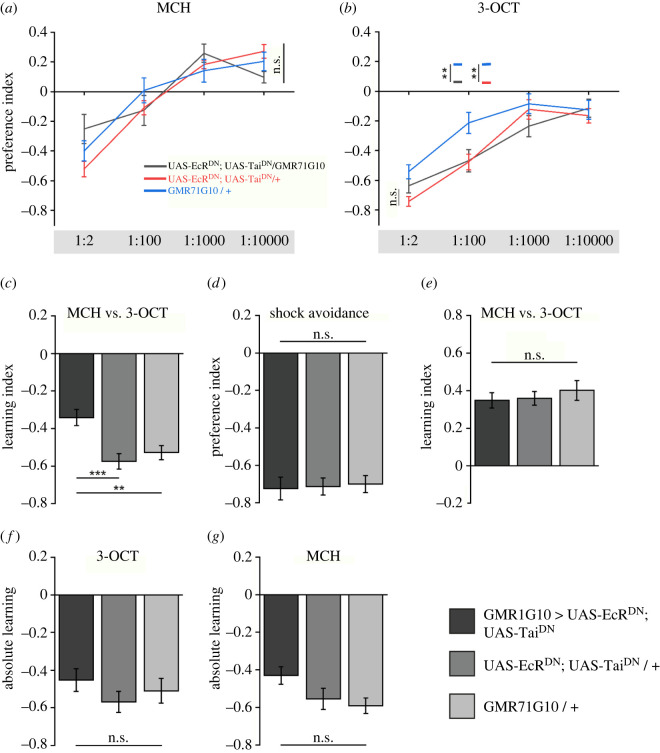
Pruning deficits of γ-KCs affect differential aversive learning, but not appetitive odour learning. (*a*) Odour preference in response to 4-methylcyclohexanol (MCH), and (*b*) in response to 3-octanol (3-OCT). The numbers below the graphs indicate odourant dilution. *n* = 8 each odour concentration. (*c*) Differential, aversive associative learning using the two odourants, MCH and 3-OCT, as conditioned stimuli. *n* = 16. (*d*) Electric shock avoidance. *n* = 14. (*e*) Differential, attractive associative learning using the two odourants, MCH and 3-OCT, as conditioned stimuli. *n* = 16. (*f*) Absolute, aversive associative learning using 3-OCT as the conditioned stimulus. *n* = 8. (*g*) Absolute, aversive associative learning using MCH as the conditioned stimulus. *n* = 8. Bars and line diagrams indicate means ± SEM. n.s. *p* > 0.05; **p < 0.01; ***p < 0.001.

Because the mushroom body of the insect brain is a neuronal circuit critically involved in associative learning and memory [11,12], we examined whether olfactory associative learning was influenced by deficits in axonal/dendritic pruning of γ-KCs. In a typical differential, aversive olfactory conditioning experiment, one odour (CS+) is temporally paired with an electric shock as a punishment (unconditioned stimulus, US). A second odour (CS−) is presented without punishment [23]. Thereby, the animals learn to avoid the CS+ and, to some extent, to approach the CS− in a T-maze test situation [32]. The odourants used here were strongly aversive at high concentrations and became less aversive or, in the case of 3-octanol, even slightly attractive at higher dilutions. Impairing mushroom body remodeling did not affect the behavioural response toward, and valence of, the odour stimuli (figure 3*a,b*). Avoidance of 4-methylcyclohexanol across several magnitudes of dilutions was not significantly different between flies with impaired γ-lobe remodelling (figure 3*a*). For the odourant 3-octanol, only the parental Gal4-strain showed a slightly, but statistically significant lower aversion of the odourant at a single dilution (1:100), but the UAS-strain was not different from the test group (figure 3*b*). However, preventing remodelling of the mushroom body caused a statistically significant, but overall mild impairment in aversive, differential odour learning (figure 3*c*). This reduction was not due to impaired detection of the electric shocks, as avoidance was not affected *per se* (figure 3*d*). On the contrary, no learning deficit occurred in an appetitive differential odour learning paradigm in which one odour (CS+) was temporally paired with a sugar reward, and a second odour was presented without any reward (CS−) (figure 3*e*). Mushroom body remodelling appears to be more important for aversive learning using punishment as reinforcer, than for appetitive learning using reward as reinforcer. However, in the case of aversive conditioning, learning was not completely abolished but was only slightly reduced.

Interestingly, no statistically significant deficit was observed in a training situation in which only one odour (CS) was paired with a punishing electric shock (figure 3*f,g*). In a test situation, this odour was presented from one side of the T-maze against a mineral oil solvent from the opposite side. In such a non-differential, absolute conditioning regime one cannot distinguish between non-associative and associative learning effects, i.e. potential changes in innate odour avoidance caused by a sensitizing, alerting electric shock, and effects induced by a true CS-US association. At the 1:100 concentration used, the odourant 3-octanol was already highly aversive, and odourant aversion after training did not exceed innate values (figure 3*b*). However, for the odourant 4-methylcyclohexanol, which was neutral at the given concentration of 1:100, absolute training caused much stronger odour avoidance (figure 3*a*). Regardless of whether learned avoidance includes non-associative, sensitizing effects, no statistically significant difference was detected between flies with impaired γ-KC remodelling and the control genotypes (figure 3*f,g*). Thus, in an absolute conditioning scenario in which the animals are not forced to differentiate between two odours, γ-KC remodelling is dispensable.

## 4. Discussion

In conclusion, impaired pruning and regrowth of γ-KCs neither affected odour avoidance nor a learning-induced change thereof. However, it was involved in learning-induced discrimination of two trained odours. Moreover, this effect was restricted to aversive learning. The animals' learning ability was not impaired by preventing the larval mushroom body from undergoing remodelling. Rather, very specific types of learning that involve odour discrimination appear to require the mature adult form of the mushroom body. The neuronal circuitry mediating associative olfactory learning in adult fruit flies is well characterized [35]. The KCs receive input from olfactory projection neurons and become sparsely activated by a given odour stimulus [36]. Dopaminergic neurons, also known as MBINs, that innervate distinct compartments along the parallel organized KC axons of all KC types mediate reinforcing valence signals of aversive stimuli, like electric shocks [37], or appetitive stimuli, like sugar rewards [38]. There is good evidence that the acquisition process and subsequent formation of short-term memories is mediated by KCs of the γ-lobes [25,26]. Dopaminergic MBINs signalling punishment innervate the axonal KC compartments *γ*1 and *γ*2, and those signalling reward innervate *γ*4 and *γ*5 [39]. The MBONs, whose dendritic trees are also confined to the respective axonal compartments, induce behavioural avoidance or attraction [39]. Temporal pairing of an odour stimulus, and thereby induced KC activity, with a valence signal, and thereby dopamine release, causes decorrelation of synaptic outputs of KC and synaptic depression of KC-to-MBON synapses at the respective compartments [40,41]. Our data indicate that this complex, overall connectivity that enables the animals to conduct associative learning is established in the adult fly even if γ-KC remodelling during pupation is prevented; the persistent larval γ-KCs appear to be largely functional in the adult animals. An alternative explanation for the relatively mild learning deficits observed could be potential plasticity during the developmental, pupal stages; that is, the *α*/β- and *α*′/*β*′-KCs might take on the role of dysfunctional γ-KCs and potentially dysfunctional synaptic connections of extrinsic γ-lobe neurons. However, we also point out that the phenotype induced by overexpressing EcR^DN^ and Tai^DN^ is not entirely complete. Therefore, we cannot rule out that a few normally remodelled γ-KCs exist which might be sufficient for the formation and functionality of an adult-like circuit.

*Drosophila* larvae can learn to associate odours with positive or negative reinforcers [24], and the larval brain connectivity underlying this ability is very similar to that of adults, but the cell numbers are lower [19,42]. In fact, *Drosophila* larvae perform well in reward-based, appetitive olfactory conditioning with the involvement of KC of embryonic origin [43]. The projection neuron-to-KC connectivity is less complex in terms of cell numbers [42], suggesting that larvae might be able to learn to differentiate fewer odourants through discriminative associative conditioning. The number of dopaminergic MBINs is also lower, but distinct reward-signalling and punishment-signalling MBINs have been identified that innervate the larval γ-lobes in a similarly compartmentalized manner [44,45]. The same applies to behaviour-instructing MBONs [44,45]. During pupation and the accompanying mushroom body remodelling, the overall larval connectivity that mediates associative olfactory learning is transformed into the adult circuit. Our data indicate that both aversive and appetitive associative learning is still possible when the axons and dendrites of larval γ-KCs persist throughout metamorphosis. But the difference in the degree to which aversive and appetitive learning is affected might indicate that reward-mediating larval dopaminergic MBINs persist and are sufficient to mediate reward learning at the adult stage, whereas larval dopaminergic MBINs and/or MBONs that mediate punishment do not. Interestingly, the overall spatial arrangement of MBONs and MBINs that mediate aversive or appetitive learning differs between larvae and adult flies. In larvae, aversive learning and short-term memory formation can be attributed to the vertical lobes of the larval mushroom body [45], but to the proximal parts of the horizontal γ-lobes in adults (*γ*1 compartment or ‘heel’ region) [39,46]. On the contrary, both in larvae and adult flies, appetitive learning and short-term memory formation can be attributed to compartments at the tips of the medial lobes [39,44,45]. This difference might perhaps explain why aversive learning is partially impaired, but appetitive learning remains intact. A recent study [47] shows that dopaminergic MBINs in larvae are of different cellular identity than the adult ones: during metamorphosis they either die or trans-differentiate into neurons innervating other brain regions. For MBONs, the situation is more complicated. Larval MBONs remodel during pupation and either shift to different mushroom body lobe compartments, or they remodel and remain at topologically similar compartments [47]. Given the profound rearrangements also at the MBIN and MBON level, we conclude that a persistent larval γ-lobe suffices to establish a functional connectivity for adult appetitive learning, and partially for aversive learning. It is important to note that also aversive learning is only mildly affected by preventing KCs from remodelling, and only in a discriminative learning task. However, appetitive discriminative learning remains unaffected by preventing KC remodelling, which indicates that the animals' general ability to discriminate the two trained odourants is still intact. A critical step in further understanding the impact of mushroom body remodelling on behaviour will be to thoroughly characterize the entire complex neuronal circuit during normal development and when remodelling is inhibited. Altering the remodelling of γ-KCs clearly affects the remodelling of at least one mushroom body extrinsic neuron, namely the anterior paired lateral neuron [48]. Clarifying how inhibiting γ-KC remodelling affects the development of the many mushroom body extrinsic MBINS and MBONs might allow us to raise more refined hypotheses on the function of Kenyon cell pruning and regrowth to be tested experimentally.

It is well known that the mushroom body negatively affects overall locomotor activity. Mutants with structurally impaired mushroom bodies, chemically ablated mushroom bodies and overexpression of tetanus toxin in mushroom bodies show increased locomotor activity [15]. Interestingly, these animals appear to have problems in terminating walking bouts [15]. Early studies on the function of insect mushroom bodies involving ablation or electrical stimulation revealed an inhibitory role of mushroom bodies on behavioural activity such as singing in crickets [49]. Currently, more refined circuit models consider the mushroom body to organize an appropriate selection of behaviours based on learned and intrinsic, motivational factors. Impairment of such behavioural selection processes could lead to increased, unmotivated and undirected behavioural activity. Our findings indicate that not only the mushroom body but also proper mushroom body remodelling are required to prevent such hyperactivity.

Impairing mushroom body remodelling affects memory formation after courtship conditioning [21], a behavioural task that is restricted to adult flies. Locomotion behaviour is also drastically different between crawling larvae and walking flies. Therefore, neuronal remodelling might be required for those behaviours and the respective adult brain circuits that are not yet present in larval brains. By contrast, it is possible that KC remodelling is not critical for establishing the circuits controlling behaviour present in both larval and adult *Drosophila*, like associative olfactory learning. It might be interesting to test in future experiments whether associative learning of sensory stimuli that are detected with higher accuracy in adult flies, but only rudimentary present in larvae, requires KC remodelling. For example, associative colour discrimination learning [13] has been reported only for adult flies, and this requires a specific γ-type KC subtype [50]. It might be interesting to test in future studies whether or not KC remodelling establishes specifically neuronal circuits required for associative learning of such adult-specific sensory modalities.

## Data Availability

All raw and meta-data underlying this publication are available at https://for2705.de/archive/4/100.

## References

[1] Schuldiner O, Yaron A. 2015 Mechanisms of developmental neurite pruning. Cell Mol. Life Sci. **72**, 101-119. (10.1007/s00018-014-1729-6) 25213356 PMC5086088

[2] Technau G, Heisenberg M. 1982 Neural reorganization during metamorphosis of the corpora pedunculata in *Drosophila melanogaster*. Nature **295**, 405-407. (10.1038/295405a0) 6799834

[3] Yaniv SP, Schuldiner O. 2016 A fly's view of neuronal remodeling. WIREs Dev. Biol. **5**, 618-635. (10.1002/wdev.241) PMC5086085 27351747

[4] Yu F, Schuldiner O. 2014 Axon and dendrite pruning in *Drosophila*. Curr. Opin. Neurobiol. **27**, 192-198. (10.1016/j.conb.2014.04.005) 24793180 PMC5086084

[5] Sugie A, Marchetti G, Tavosanis G. 2018 Structural aspects of plasticity in the nervous system of *Drosophila*. Neural Dev. **13**, 14. (10.1186/s13064-018-0111-z) 29960596 PMC6026517

[6] Venken KJT, Simpson JH, Bellen HJ. 2011 Genetic manipulation of genes and cells in the nervous system of the fruit fly. Neuron **72**, 202-230. (10.1016/j.neuron.2011.09.021) 22017985 PMC3232021

[7] Eichler K et al. 2017 The complete connectome of a learning and memory centre in an insect brain. Nature **548**, 175-182. (10.1038/nature23455) 28796202 PMC5806122

[8] Takemura S et al. 2017 A connectome of a learning and memory center in the adult *Drosophila* brain. Elife **6**, e26975. (10.7554/eLife.26975) 28718765 PMC5550281

[9] Zheng Z et al. 2020 A complete electron microscopy volume of the brain of adult *Drosophila melanogaster*. Cell **174**, 730-743. (10.1016/j.cell.2018.06.019) PMC6063995 30033368

[10] Li F et al. 2020 The connectome of the adult *Drosophila* mushroom body provides insights into function. Elife **9**, e62576. (10.7554/eLife.62576) 33315010 PMC7909955

[11] Zars T. 2000 Behavioral functions of the insect mushroom bodies. Curr. Opin. Neurobiol. **10**, 790-795. (10.1016/S0959-4388(00)00147-1) 11240291

[12] Heisenberg M. 2003 Mushroom body memoir: from maps to models. Nat. Rev. Neurosci. **4**, 266-275. (10.1038/nrn1074) 12671643

[13] Vogt K, Schnaitmann C, Dylla KV, Knapek S, Aso Y, Rubin GM, Tanimoto H. 2014 Shared mushroom body circuits underlie visual and olfactory memories in *Drosophila*. Elife **3**, e02395. (10.7554/eLife.02395) 25139953 PMC4135349

[14] McBride SM, Giuliani G, Choi C, Krause P, Correale D, Watson K, Baker G, Siwicki KK. 1999 Mushroom body ablation impairs short-term memory and long-term memory of courtship conditioning in *Drosophila melanogaster*. Neuron **24**, 967-977. (10.1016/s0896-6273(00)81043-0) 10624959

[15] Martin JR, Ernst R, Heisenberg M. 1998 Mushroom bodies suppress locomotor activity in *Drosophila melanogaster*. Learn Mem. **5**, 179-191. (10.1101/lm.5.1.179) 10454382 PMC311252

[16] Joiner WJ, Crocker A, White BH, Sehgal A. 2006 Sleep in *Drosophila* is regulated by adult mushroom bodies. Nature **441**, 757-760. (10.1038/nature04811) 16760980

[17] Tsao CH, Chen CC, Lin CH, Yang HY, Lin S. 2016 *Drosophila* mushroom bodies integrate hunger and satiety signals to control innate food-seeking behavior. Elife **7**, e35264. (10.7554/eLife.35264) PMC5910021 29547121

[18] Siju KP, Štih V, Aimon S, Gjorgjieva J, Portugues R, Grunwald Kadow IC. 2020 Valence and state-dependent population coding in dopaminergic neurons in the fly mushroom body. Curr. Biol. **30**, 2104-2115. (10.1016/j.cub.2020.04.037) 32386530

[19] Thum A, Gerber B. 2019 Connectomics and function of a memory network: the mushroom body of larval *Drosophila*. Curr. Opin. Neurobiol. **54**, 146-154. (10.1016/j.conb.2018.10.007) 30368037

[20] Lee T, Lee A, Luo L. 1999 Development of the *Drosophila* mushroom bodies: sequential generation of three distinct types of neurons from a neuroblast. Development **126**, 4065-4076. (10.1242/dev.126.18.4065) 10457015

[21] Redt-Clouet C, Trannoy S, Boulanger A, Tokmatcheva E, Savvateeva-Popova E, Parmentier ML, Preat T, Dura JM. 2012 Mushroom body neuronal remodelling is necessary for short-term but not for long-term courtship memory in *Drosophila*. Eur. J. Neurosci. **35**, 1684-1691. (10.1111/j.1460-9568.2012.08103.x) 22571719

[22] Tempel BL, Bonini N, Dawson DR, Quinn WG. 1983 Reward learning in normal and mutant *Drosophila*. Proc. Natl Acad. Sci. USA **80**, 1482-1486. (10.1073/pnas.80.5.1482) 6572401 PMC393622

[23] Tully T, Quinn WG. 1985 Classical conditioning and retention in normal and mutant *Drosophila melanogaster*. J. Comp. Physiol. A **157**, 263-277. (10.1007/BF01350033) 3939242

[24] Widmann A, Eichler K, Selcho M, Thum AS, Pauls D. 2018 Odor-taste learning in *Drosophila* larvae. J. Insect Physiol. **106**, 47-54. (10.1016/j.jinsphys.2017.08.004) 28823531

[25] Zars T, Fischer M, Schulz R, Heisenberg M. 2000 Localization of a short-term memory in *Drosophila*. Science **288**, 672-675. (10.1126/science.288.5466.672) 10784450

[26] Qin H, Cressy M, Li W, Coravos JS, Izzi SA, Dubnau J. 2012 Gamma neurons mediate dopaminergic input during aversive olfactory memory formation in *Drosophila*. Curr. Biol. **22**, 608-614. (10.1016/j.cub.2012.02.014) 22425153 PMC3326180

[27] Pfeiffer BD et al. 2008 Tools for neuroanatomy and neurogenetics in *Drosophila*. Proc. Natl Acad. Sci. USA **105**, 9715-9720. (10.1073/pnas.0803697105) 18621688 PMC2447866

[28] Cherbas L, Hu X, Zhimulev I, Belyaeva E, Cherbas P. 2003 EcR isoforms in *Drosophila*: testing tissue-specific requirements by targeted blockade and rescue. Development **130**, 271-284. (10.1242/dev.00205) 12466195

[29] Jang AC, Chang YC, Bai J, Montell D. 2009 Border-cell migration requires integration of spatial and temporal signals by the BTB protein Abrupt. Nat. Cell Biol. **11**, 569-579. (10.1038/ncb1863) 19350016 PMC2675665

[30] Chiu JC, Low KH, Pike DH, Yildirim E, Edery I. 2010 Assaying locomotor activity to study circadian rhythms and sleep parameters in *Drosophila*. J. Vis. Exp. **43**, 2157. (10.3791/2157) PMC3229366 20972399

[31] Schwaerzel M, Monastirioti M, Scholz H, Friggi-Grelin F, Birman S, Heisenberg M. 2003 Dopamine and octopamine differentiate between aversive and appetitive olfactory memories in *Drosophila*. J. Neurosci. **23**, 10 495-10 502. (10.1523/JNEUROSCI.23-33-10495.2003) PMC6740930 14627633

[32] Barth J, Dipt S, Pech U, Hermann M, Riemensperger T, Fiala A. 2014 Differential associative training enhances olfactory acuity in *Drosophila melanogaster*. J. Neurosci. **34**, 1819-1837. (10.1523/JNEUROSCI.2598-13.2014) 24478363 PMC6827587

[33] Alyagor I, Berkun V, Keren-Shaul H, Marmor-Kollet N, David E, Mayseless O, Issman-Zecharya N, Amit I, Schuldiner O. 2018 Combining developmental and perturbation-seq uncovers transcriptional modules orchestrating neuronal remodeling. Dev. Cell **47**, 38-52.e6. (10.1016/j.devcel.2018.09.013) 30300589 PMC6179959

[34] Shaw PJ, Cirelli C, Greenspan RJ, Tononi G. 2000 Correlates of sleep and waking in *Drosophila melanogaster*. Science **287**, 1834-1837. (10.1126/science.287.5459.1834) 10710313

[35] Boto T, Stahl A, Tomchik SM. 2020 Cellular and circuit mechanisms of olfactory associative learning in *Drosophila*. J. Neurogenet. **34**, 36-46. (10.1080/01677063.2020.1715971) 32043414 PMC7147969

[36] Turner GC, Bazhenov M, Laurent G. 2008 Olfactory representations by *Drosophila* mushroom body neurons. J. Neurophysiol. **99**, 734-746. (10.1152/jn.01283.2007) 18094099

[37] Riemensperger T, Völler T, Stock P, Buchner E, Fiala A. 2005 Punishment prediction by dopaminergic neurons in *Drosophila*. Curr. Biol. **15**, 1953-1960. (10.1016/j.cub.2005.09.042) 16271874

[38] Liu C et al. 2012 A subset of dopamine neurons signals reward for odour memory in *Drosophila*. Nature **488**, 512-516. (10.1038/nature11304) 22810589

[39] Aso Y et al. 2014 The neuronal architecture of the mushroom body provides a logic for associative learning. Elife **3**, e04577. (10.7554/eLife.04577) 25535793 PMC4273437

[40] Hige T, Aso Y, Modi MN, Rubin GM, Turner GC. 2015 Heterosynaptic plasticity underlies aversive olfactory learning in *Drosophila*. Neuron **88**, 985-998. (10.1016/j.neuron.2015.11.003) 26637800 PMC4674068

[41] Bilz F, Geurten BRH, Hancock CE, Widmann A, Fiala A. 2020 Visualization of a distributed synaptic memory code in the *Drosophila* brain. Neuron **106**, 963-976.e4. (10.1016/j.neuron.2020.03.010) 32268119

[42] Gerber B, Stocker RF. 2007 The *Drosophila* larva as a model for studying chemosensation and chemosensory learning: a review. Chem. Senses **32**, 65-89. (10.1093/chemse/bjl030) 17071942

[43] Pauls D, Selcho M, Gendre N, Stocker RF, Thum AS. 2010 *Drosophila* larvae establish appetitive olfactory memories via mushroom body neurons of embryonic origin. J. Neurosci. **30**, 10 655-10 666. (10.1523/JNEUROSCI.1281-10.2010) PMC6634688 20702697

[44] Saumweber T et al. 2018 Functional architecture of reward learning in mushroom body extrinsic neurons of larval *Drosophila*. Nat. Commun. **9**, 1104. (10.1038/s41467-018-03130-1) 29549237 PMC5856778

[45] Eschbach C et al. 2020 Recurrent architecture for adaptive regulation of learning in the insect brain. Nat. Neurosci. **23**, 544-555. (10.1038/s41593-020-0607-9) 32203499 PMC7145459

[46] Hancock CE, Rostami V, Rachad EY, Deimel SH, Nawrot MP, Fiala A. 2022 Visualization of learning-induced synaptic plasticity in output neurons of the *Drosophila* mushroom body γ-lobe. Sci. Rep. **12**, 10421. (10.1038/s41598-022-14413-5) 35729203 PMC9213513

[47] Truman JW, Price J, Miyares RL, Lee T. 2022 Metamorphosis of memory circuits in *Drosophila* reveal a strategy for evolving a larval brain. *bioRxiv* 2022.06.09.495452. (10.1101/2022.06.09.495452)

[48] Mayseless O, Berns DS, Yu XM, Riemensperger T, Fiala A, Schuldiner O. 2018 Developmental coordination during olfactory circuit remodeling in *Drosophila*. Neuron **99**, 1204-1215.e5. (10.1016/j.neuron.2018.07.050) 30146303

[49] Huber F. 1962 Central nervous control of sound production in crickets and some speculations on its evolution. Evolution **16**, 429-442. (10.2307/2406177)

[50] Vogt K, Aso Y, Hige T, Knapek S, Ichinose T, Friedrich AB, Turner GC, Rubin GM, Tanimoto H. 2016 Direct neural pathways convey distinct visual information to *Drosophila* mushroom bodies. Elife **5**, e14009. (10.7554/eLife.14009) 27083044 PMC4884080

